# Microleakage Evaluation of Two Methacrylate-Based Composites (GC Kalore and Luna SDI) in Class II Restorations: A Laboratory Study

**DOI:** 10.1155/2022/3835694

**Published:** 2022-12-28

**Authors:** Kooshan Moradi, Sadaf Sadat Mahmoudinezhad, Mehran Mapar

**Affiliations:** Department of Operative Dentistry, School of Dentistry, Ahvaz Jundishapur University of Medical Sciences, Ahvaz, Iran

## Abstract

**Objective:**

In recent years, dental composite resins such as tooth-colored restoration are frequently used to restore dental cavities, coronal fractures, and congenital defects. This study aimed to evaluate the microleakage of two methacrylate-based composites (GC Kalore and Luna SDI) in class II restorations.

**Materials and Methods:**

In this experimental study, a total of 18 intact human premolars previously extracted for periodontal and orthodontic reasons were randomly divided into two groups. Similar class II cavities (box only) were prepared on all teeth and restored with two different composites. In group 1, a bonding agent (Single Bond 2-SB2; 3M ESPE) and Luna SDI composite in mesial cavities and GC Kalore composite in distal cavities were used. In group 2, Single Bond 2 and GC Kalore composite in mesial cavities and Luna SDI composite in distal cavities were applied. They were then subjected to 2000 thermal cycles in a water bath between 5–55°C (dwell time: 30 seconds in every bath and transfer time: 10 seconds). Then, they were immersed in a 2% basic fuchsin dye solution for 24 hours. After rinsing with water, they were sectioned mesiodistally and evaluated for microleakage using a stereomicroscope.

**Results:**

Independent *t*-test (Mann–Whitney test) showed no statistically significant difference for microleakage in mesial and distal class II restorations between GC Kalore composite and Luna SDI composite (*p* = 1.000) (*p*= 0.852). A total of 83.4% of the Luna SDI composite samples and 66.6% of the GC Kalore composite had a microleakage score of ≤3 in class II cavities.

**Conclusion:**

In the present study, marginal microleakage was found mainly at the gingival floor extending to 1/3 of the axial wall for the Luna SDI composite and GC Kalore composite. Furthermore, no statistically significant difference was found between the microleakage of the Class II cavities restored with Luna SDI composite and GC Kalore composite.

## 1. Introduction

In recent years, dental composite resins such as tooth-colored restoration are frequently used to restore dental cavities, coronal fractures, and congenital defects [1]. These materials are technically sensitive and brittle, and effective marginal adaption is crucial for their clinical success. Lower cavity configuration (C factor), physical and chemical properties of composite resins and bonding materials, the incremental method for packing composite resins, and modification in resin matrix formulation seems to be various factors involved in increasing marginal adaption, reducing polymerization stress, and reducing microleakage [2, 3]. Marginal staining, cuspal deflection, mechanical failure, and recurrent caries are the most common outcomes of marginal microleakage [4]. Breaking the carbon-carbon double bonds after the polymerization of composite resins results in shrinkage and accumulation of stress within the material and cavity walls, which leads to a gap in this area [5]. GC Kalore composite is a low-shrinkage nanocomposite containing DuPont monomer (a high molecular weight urethane methacrylate-based monomer with fewer C=C double bonds) [6]. Luna SDI is another nanohybrid composite with long-chain cross-linking monomers intended to reduce intermonomer distance within a polymer compared to shorter cross-linking monomers [7].

To the best of our knowledge, no study has evaluated the marginal microleakage of the Luna SDI composite. The current study aimed to compare the microleakage of two nanohybrid methacrylate-based resin composites (GC Kalore composite and Luna SDI composite) in class II restorations of premolar teeth. The null hypothesis of the study was that there is no significant difference in microleakage of the Luna SDI composite and GC Kalore composite.

## 2. Materials and Methods

In the present experimental study, a total of 18 human premolar teeth were used. The premolars were extracted for periodontal and orthodontic reasons. The inclusion criteria for teeth were as follows: intact crown without crack, caries, and restorations. The samples were cleaned with Pumice and distilled water, disinfected with hypochlorite (0.5%) for 5 minutes and stored in normal saline at room temperature until the study was carried out.

### 2.1. Cavity Preparation

36 standardized Class II cavities with gingival margins placed 1 mm below the cementoenamel junction were made on the mesial and distal surfaces using a diamond fissure bur (No 837L/010; Tizacavan, Tehran, Iran) with high-speed handpiece underwater spray. A new bur was used for every preparation in order to maintain cutting efficiency. All the cavity dimensions were standardized as follows: 4 mm occlusogingival height, 4 mm buccolingual width, and axial wall 2 mm from the mesial and distal tooth surface. All the cavities were prepared by a single operator. Cavity dimensions were verified with a digital caliper.

### 2.2. Restorative Procedure

Materials used in this paper are listed in Table 1. Samples were etched with 37% phosphoric acid gel, then rinsed and air-dried, and impregnated with a bonding agent (Single Bond 2- SB2; 3M ESPE; St Paul, MN, USA). In order to eliminate the probable effect of the anatomical difference between mesial and distal surfaces of premolar teeth on the amount of microleakage, we considered two groups with different filling patterns: the type of the composite in distal cavities of the first group was the same as the type of the composite in mesial cavities in the second group. In addition, the type of the composite in the mesial cavities of the first group was the same as the type of the composite in the distal cavities of the second group. All the composites were applied using the incremental filling technique after adjusting a circumferential metal matrix around the tooth. A LED light curing unit (Woodpecker, Shanghai, China) with a light intensity of 1000 mW/cm^2^ was used for curing. The curing time for bonding was 20 seconds and for the composites was 30 seconds according to the manufacturer's instructions. The light guide was as close as possible to the restoration surface.

Samples were randomly divided into two groups (*n* = 9) as follows:

  Group 1: mesial cavities were filled with Luna SDI composite (SDI, Vic, Australia), and distal cavities were filled with GC Kalore composite (GC, Tokyo, Japan)
  Group 2: distal cavities were filled with Luna SDI composites, and mesial cavities were filled with GC Kalore composites

### 2.3. Thermocycling Procedure

The samples were mounted up to 1mm apical to cervical margins of restorations in self-curing acrylic resin (Acropars, Iran). A small blue dot was drawn at the mesial side of the half of the specimens and a small red dot at the distal side of the others to eliminate the impact of the anatomical variation at the mesial and distal surfaces of the teeth. They were then subjected to 2000 thermal cycles by a thermocycling machine in a water bath between 5 and 55°C (dwell time: 30 seconds in every bath and transfer time: 10 seconds) (Baradaran Pouya, Iran) [8].

### 2.4. Staining of the Samples

All the tooth surfaces, except the restoration and a 1 mm zone adjacent to the margins of the restorations, were coated with two layers of nail varnish (Anny, 60 seconds, Germany). Then, they were submerged in a 2% basic fuchsin dye solution for 24 hours. The teeth were then rinsed with running water and sectioned mesiodistally along the center of each restoration. Each part was visualized under a stereomicroscope at ×16 magnifications to assess microleakage.

### 2.5. Microleakage Analysis

The degree of dye penetration was scored as follows [9, 10]:

  Score 0: no dye penetration
  Score 1: dye penetration extending to the external 1/2 of the gingival floor
  Score 2: dye penetration extending to the internal 1/2 of the gingival floor without reaching the axial walls
  Score 3: dye penetration from the gingival floor up to 1/3 axial walls
  Score 4: dye penetration from the gingival floor up to 2/3 axial walls
  Score 5: dye penetration was present along the axial wall and gingival floor

### 2.6. Statistical Analysis

The results were statistically analyzed using SPSS V22.0. The *p* value <0.05 was considered as the significant level. Independent *t*-test (Mann–Whitney test) were used for comparing the microleakages. The methodology was reviewed by an independent statistician.

## 3. Results

**Figure 1:**
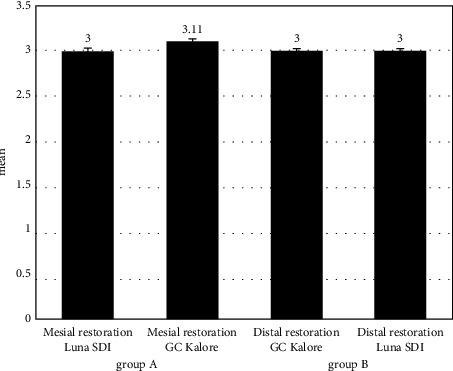
The comparison of the microleakage of each composite between mesial and distal class II restorations regarding their group.

The frequency of different scores of microleakage is shown in Table 2.  Figure 1 showed the comparison of the microleakage of each composite between mesial and distal class II restorations regarding their group. According to the Independent *t*-test (Mann–Whitney test), there was no statistically significant difference between the microleakage in mesial class II restorations with Luna SDI composite (Mean ± SD = 3 ± 0.71) and the microleakage in distal class II restorations with GC Kalore composite (Mean ± SD = 3.11 ± 1.54) in group A (*p* = 0.875). Besides, no statistically significant difference was found in mesial class II restorations with GC Kalore composite (Mean ± SD = 3 ± 1.41) and the microleakage in distal class II restorations with Luna SDI composite (Mean ± SD = 3 ± 0.87) in group B (*p* = 1.000), Independent *t*-test (Mann–Whitney test) showed no statistically significant difference for microleakage in mesial class II restorations between GC Kalore composite and Luna SDI composite (*p* = 1.000). There was no statistically significant difference for distal class II restorations, respectively, (*p* = 0.852) (Table 3).

No statistically significant difference was found between mesial and distal class II restorations for GC Kalore composite regarding the composite type (*p* = 0.875). Also, we found no statistically significant difference between mesial and distal class II restorations for Luna SDI composite, respectively, (*p* = 1.000) (Table 4).

## 4. Discussion

The marginal sealing is a critical factor for the success of the composite restorations. It should be considered to avoid pulpal irritations, cuspal deflection, recurrent caries, and marginal staining [11–13]. The restriction of the resin composite to contract freely after polymerization leads to shrinkage stress on the surrounding tooth structure [14]. The manufacturers of the latest species of composites (nanocomposites and nanohybrid composites) try to prove the ability of their product to maintain marginal integrity and low shrinkage stress over the years. Assessment of the microleakage is a common method to compare the quality of the restorative materials [15–18]. The present study compared the marginal microleakage of two methacrylate-based nanohybrid composites (GC Kalore and Luna SDI) in mesial and distal class II cavities in 18 premolars (36 class II restorations in mesial and distal of tooth). In order to evaluate the marginal shrinkage of the two mentioned composites, all the cavities were restored using the incremental method with the same bonding agent (Single Bond 2- SB2) and the same cavity configuration factor (C factor) and cured with the same light cure device. Despite a high C factor for class II cavities, in distal cavities, 66.7% of the samples of Luna SDI composite and 44.4% of the samples of GC Kalore showed a microleakage of score 3. As much as 55.6% of the samples of Luna SDI composite and 44.4% of the samples of GC Kalore composite demonstrated a microleakage of score 3 in mesial cavities, respectively. The null hypothesis of the study that there is no significant difference in microleakage of the Luna SDI composite and GC Kalore composite is confirmed (*p* > 0.05).

A resin composite is composed of four major components: organic polymer matrix, inorganic filler particles, coupling agent, and the initiator-accelerator system. The technology of methacrylate-based nanohybrid composites is based on the utilization of dimethacrylates as the monomer instead of methylmethacrylate (MMA) previously used in some early products [19]. Bisphenol A-glycidyl methacrylate (Bis-GMA) and urethane dimethacrylate (UDMA) monomers are highly viscous fluids because of their high molecular weights, which are the reasons for the low marginal shrinkage of Luna SDI composite. The high molecular weight monomer of GC Kalore composite known as DX-511, licensed from Dupont, prevents the polymerization shrinkage of the GC Kalore composite with its long rigid core. Nevertheless, the flexible end groups promote reactivity, enhance monomer-polymer conversion, and compensate for the reduced reactivity usually associated with long monomer chains [20]. Several studies evaluate the marginal adaptation of GC Kalore composite. Juloski et al. [21] evaluated the microleakage of Class II cavities restored with SureFil SDR flow, Filtek Silorane, G-aenial Flo bulk fill, G-aenial Universal Flo bulk fill, and GC Kalore bulk-fill composites. Microleakage was not observed at the enamel interface in any group by scoring the depth of silver-nitrate penetration. At the dentin interface, the microleakage of G-aenial Flo bulk fill, G-aenial Universal Flo bulk fill, and GC Kalore composite recorded insignificantly different and lower than SureFil SDR flow. In line with the previous study, Shibasaki et al. [22] found that the smaller number of carbon-carbon double bounds in DX-511 leads to lower marginal shrinkage for the GC Kalore composite.

Based on the manufacturers' claim, the molecular weight of DX-511 is twice the molecular weight of Bis-GMA, which may result in lower polymerization shrinkage. GC Kalore composite does not have Bis-GMA. Since our study showed no significant difference between the microleakage of Luna SDI composite restorations and GC Kalore composite restorations, it seems that the DX-511 with the higher molecular weight and Bis-GMA act similarly in reducing the polymerization shrinkage. According to the study of Bagheri et al. [23], these high molecular weight monomers may also be the reason for the high fracture toughness of the Luna SDI composite compared to the Estelite composite.

In order to overcome high viscous components resulting from their high molecular weight and better clinical manipulation, the triethylene glycol dimethacrylate (TEGDMA) molecule is used in the Luna SDI composite and GC Kalore composite.

Another solution to overcome the polymerization shrinkage and shrinkage stress is the addition of inorganic and prepolymerized nonreactive fillers in the matrix to reduce the concentration of reactive methacrylate groups, which tend to create highly crossed-linked resin after polymerization [24, 25]. For this reason, based on the manufacturers' claim, 77% of the weight of Luna SDI composite and 80% of the weight of GC Kalore composite contain large (0.4 to 5 *μ*m) and nanometer-sized filler particles. These fillers also control various aesthetic features, thermal expansion, degree of conversion, and other physical and mechanical properties of composite resins.

Takahashi et al. [24] investigated the effects of polymerization contraction, shrinkage stress, and Young's modulus of nanofiller-containing resin composites (FiltekTM Supreme XT, Grandio, Kalore, MI Flow, Tetric EvoCeram, Venus® Diamond, FiltekTM Z250, and Durafill® VS) on the early marginal adaption of restorations. GC Kalore composite and Venus Diamond composite showed consistent gap-free margins in bonded dentin cavities taking into account their high filler content, high molecular weight monomer, low stiffness, and low elastic modulus. Kermanshah et al. [26] prepared class V cavities on the facial and lingual surfaces of 48 human premolars restored with Filtek P90, Aelite LS Posterior, Grandio, and GC Kalore composites, and thermocycled them for 2000 cycles (5–55°C). They found that occlusal and gingival microleakage were not affected by cyclic loading in class V cavities regarding the composition of nanohybrid composites. In addition, Hoseinifar et al. [27] evaluated the clinical performance of a packable composite (Filtek P60) and a low shrinkage methacrylate-based composite (GC Kalore) after one year on 50 class I or II restorations in 25 patients. Each patient received two restorations. All the restorations were examined for marginal adaptation, marginal staining, secondary caries, and postoperative sensitivity after one week, six months, and one year according to the modified United States Public Health Service (USPHS) criteria. Results showed equally good clinical performance for GC Kalore composite and Filtek P60 composite. Our study support previous results for GC Kalore since 55.5% of the restorations in mesial and distal cavities showed a microleakage score of ≤3.

To the best of our knowledge, no study has investigated the microleakage of Luna SDI composite. The manufacturers of Luna SDI composite claim that the volumetric shrinkage of this composite is 2.88%. The present study revealed that 77.8% of the Luna SDI composite samples had a microleakage score of ≤3 in mesial cavities, and 88.9% of the samples had a microleakage score of ≤3 in distal cavities which support the mentioned manufacturers' claim.

The present study had the limitation of measuring the degree of conversion, which refers to the thickness of polymerized composite resins. Furthermore, the particles of the fushin dye are small, and the restorations may reveal overestimated microleakage during thermocycling. Furthermore, in vitro and clinical studies are recommended regarding the measurement of fracture toughness and flexural strength, which are the other physical properties determining the efficiency of dental materials.

Marginal microleakage was found mainly at the gingival floor extending to 1/3 of the axial wall for Luna SDI composite and GC Kalore composite. Furthermore, no statistically significant difference was found between the microleakage of the Class II cavities restored with Luna SDI composite and GC Kalore composite. Further experimental and clinical studies are needed to compare the efficiency of the materials analyzed in the present study.

## Figures and Tables

**Table 1:** Materials used in this study.

	Manufacturer	Composition
GC Kalore	GC Corporation, Tokyo, Japan	DX-511, UDMA, dimethacrylate comonomers, prepolymerized filler(with 100 nm lanthanoid fluoride), 700 nm fluoro-alumino-silicate glass, 700 nm strontium/barium glass, 16 nm silicon dioxide, lanthanoid fluoride
Luna	SDI, Viv, Australia	UDMA/Bis-GMA/TEGDMA Strontium alumino silicate, amorphous silica.0.2–2 *μ*m 200–400 nm

**Table 2:** Distribution of microleakage scores for two methacrylate-based composite (Luna SDI and GC Kalore).

Group	Cavity	Composite	*n*	Microleakage score					
				0	1	2	3	4	5
A	M	Luna SDI	9	0 (0%)	0 (0%)	(22.2%) 2	5 (55.6%)	(22.2%) 4	0 (0%)
	D	GC kalore	9	(11.1%) 1	0 (0%)	(11.1%) 1	(44.4%) 4	(11.1%) 1	(22.2%) 2

B	M	GC kalore	9	(11.1%) 1	0 (0%)	(11.1%) 1	(44.4%) 4	(22.2%) 2	(11.1%) 1
	D	Luna SDI	9	0 (0%)	0 (0%)	(22.2%) 2	(66.7%) 6	0 (0%)	(11.1%) 1

**Table 3:** Mean and standard deviation of microleakage of two composites (Luna SDI and GC Kalore) based on mesial and distal.

Cavity	Luna SDI		GC Kalore		*p* value
	Mean ± SD	*n*	Mean ± SD	*n*	
M	3.00 ± 0.71	9	3.00 ± 1.41	9	1.000
D	3.00 ± 0.87	9	3.11 ± 1.54	9	0.852

SD = std. deviation; M = mesial; D = distal; *n* = number.

**Table 4:** Mean and standard deviation of microleakage of two composites (Luna SDI and GC Kalore) based on the composite type.

Composite	Mesial		Distal		*p* value
	*n*	Mean ± SD	*n*	Mean ± SD	
GC kalore	9	3.00 ± 1 .41	9	3.11 ± 1.54	0.875
Luna SDI	9	3.00 ± 0.71	9	3.00 ± 0.87	1.000

SD = std. deviation; *n* = number.

## Data Availability

The data used to support the findings of this study are available from the corresponding author upon request. (Contact details: sadafmahmoudinezhad@gmail.com).
